# Mesial Root Tipping of the Maxillary Lateral Incisor to Facilitate Eruption of an Impacted Canine: A Retrospective Study

**DOI:** 10.3390/diagnostics16121859

**Published:** 2026-06-16

**Authors:** Ornjira Wiriyapongsukit, Mittida Raksanaves, Chairat Charoemratrote

**Affiliations:** 1 Private Practice, Surat Thani 84000, Thailand; 2 Department of Orthodontics, Faculty of Dentistry, Chulalongkorn University, Bangkok 10330, Thailand; 3 Private Practice, Tucson, AZ 85658, USA; 4 Orthodontic Section, Department of Preventive Dentistry, Faculty of Dentistry, Prince of Songkla University, Songkhla 90112, Thailand

**Keywords:** impacted maxillary canine, maxillary lateral incisor, mesial root tipping, tooth eruption, panoramic radiography

## Abstract

**Background/Objectives**: The maxillary lateral incisor (U2) root has been proposed to influence the eruption pathway of the maxillary canine. This retrospective study aimed to evaluate the association between mesial root tipping of the U2 and the eruption of impacted maxillary canine (IPU3), and to identify radiographic predictors of eruptive movement. **Methods**: Orthopantomograms of 37 IPU3 from 29 patients aged 10–12 years were analyzed in this retrospective responder study; all included cases showed initiation of IPU3 eruption following U2 mesial root tipping, and this design was considered when interpreting potential selection bias and overestimation of effect. U2 and canine (U3) positions were measured at treatment initiation (T0) and at the 1-year follow-up (T1). Positional changes were analyzed using paired t-tests, Pearson’s correlation, and multiple linear regression. **Results**: Significant positional changes were observed for both U2 and U3 (all *p* < 0.001). The blockage point on the distal U2 root (2DBlock) shifted mesially by 2.0 mm, and U2 root angulation increased by 5.6° at the distal surface and 6.3° along its long axis. The U3 cusp tip (3Cusp) moved vertically by 3.7 mm, distally by 2.1 mm, and tipped distally by 7.5°. A strong correlation (r = 0.697) was observed between mesial root movement (2DHorz) and vertical cusp displacement (3Vert). Regression analysis identified 2DHorz as the only significant predictor of 3Vert (*p* < 0.001), explaining 51% of the variance; this indicates moderate explanatory power, while the remaining 49% suggests that additional biological, developmental, and three-dimensional spatial factors may also influence eruptive movement. **Conclusions**: Mesial root tipping of the U2 facilitates IPU3 eruption in early adolescents (10–12 years), specifically in cases with non-palpable IPU3 in sector II and fully developed U2 roots. Horizontal repositioning of the U2 root may serve as a clinically relevant radiographic indicator for guiding interceptive treatment; however, these findings should be interpreted as associations rather than evidence of causality.

## 1. Introduction

Maxillary canines (U3) are the second most frequently impacted teeth after third molars because of their long eruption path and deep developmental position. The prevalence of impacted maxillary canines (IPU3) ranges 1–2.5% [1,2,3] and occurs 2.3–3-fold more often in females, typically unilaterally [1]. IPU3 may cause adjacent tooth displacement, root resorption, and cyst formation [4,5]. As these complications are often asymptomatic, early diagnosis through clinical and radiographic evaluation is essential [6,7].

Diagnosis of IPU3 involves both clinical and radiographic assessments. Orthopantomograms (OPGs) remain the primary imaging modality because of their wide coverage and lower radiation exposure [8,9]. Although cone-beam computed tomography (CBCT) provides superior anatomical detail [4,10,11], it should be used judiciously.

Treatment options for IPU3, include observation, extraction of deciduous canines to create space, maxillary arch expansion, and surgical exposure with orthodontic traction [11,12,13]. However, when the IPU3 is located within 0.5 mm of the maxillary lateral incisor (U2) root, the risk of root resorption and iatrogenic injury increases [8,14]. In such cases, direct traction or surgical exposure may be contraindicated [1]. Mesially moving the U2 root away from the IPU3 may reduce the risk of root resorption and allow safer eruption guidance if surgery becomes necessary.

The etiology of IPU3 remains multifactorial, primarily explained by the genetic and guidance theories [1,3]. Other factors, including arch space availability, maxillary growth pattern, and genetic predisposition, may also influence the eruption pathway of the U3. The genetic theory attributes palatal impaction to hereditary factors [2], whereas the guidance theory emphasizes the role of U2 morphology and position in directing U3 eruption [15]. During the “ugly duckling” stage, the erupting U3 travels along the distal surface of the U2 root, and normal alignment occurs as spacing resolves [16]. This indicates that U2 root position plays a critical role in U3 eruption [15]. Absence of distal inclination of the U2 crown has been linked to canine impaction [17]; however, evidence supporting the guidance theory remains inconclusive. Previous studies have primarily focused on crown inclination or positional relationships of the U2 using two-dimensional radiographs, with limited evaluation of root movement dynamics during eruption. In addition, conflicting findings regarding the predictive value of U2 morphology and inclination suggest that the role of the lateral incisor in guiding canine eruption may be more complex than previously assumed. Therefore, further investigation of U2 root positioning and its relationship with IPU3 eruption is warranted. Simulating the natural mesial tipping observed in the ugly duckling stage may promote canine eruption; however, quantitative evaluation of mesial U2 root tipping during canine eruption remains limited. Therefore, the present study investigated the relationship between mesial U2 root tipping and IPU3 eruption. The specific objectives were to: (1) compare positional changes in U2 and U3 between treatment initiation (T0) and 1-year follow-up (T1); (2) assess correlation between U2 root movement and IPU3 eruption; and (3) determine whether U2 root positional changes predict the extent of U3 eruption.

## 2. Materials and Methods

This study included patients whose IPU3 eruption was initiated by mesial root tipping of the U2.

Sample size calculation was performed using G*Power software version 3.1.9.7 (Franz Faul; Christian-Albrechts-Universitat, Kiel, Germany) (f^2^ = 0.35, α = 0.05, power = 0.80) for linear multiple regression, and a minimum of 36 teeth were required to detect a significant effect. The effect size (f^2^ = 0.35) was defined as large according to Cohen.

### 2.1. Participants

Patients were retrospectively recruited from the Orthodontic Section, Faculty of Dentistry, Prince of Songkla University.

Inclusion criteria were as follows: (1) age 10–12 years; (2) high-quality OPGs at T0 and T1; (3) IPU3 diagnosed at T0 based on absence of a palpable canine bulge and OPG exhibiting U3 crown overlapping U2 root in at least sector II, according to Ericson and Kurol (sector I = no overlap; sector II = overlap of the distal half of the U2 root; sector III-V = progressive more mesial overlap) [18]; and (4) complete root formation of U2 [19].

Before treatment initiation (T0), all patients were observed for 6 months to assess spontaneous IPU3 movement. If no spontaneous eruption occurred, orthodontic treatment was initiated and defined as T0. The follow-up radiograph obtained one year later was designated as T1.

Exclusion criteria were as follows: (1) systemic diseases or medications affecting tooth movement, (2) presence of maxillary pathology, (3) peg-shaped U2, (4) congenital absence of maxillary teeth (except third molars), (5) U2 root resorption, or (6) facial trauma.

### 2.2. Orthodontic Treatment

All participants received Roth’s prescription 0.018” edgewise appliances (Ormco Corporation, Orange, CA, USA). U2 mesial root movement was achieved via angulated bracket placement (clockwise rotation on right U2; counterclockwise rotation on left U2), which generated controlled second-order moments to facilitate mesial root tipping. Leveling was performed using 0.014” nickel-titanium (NiTi) and 0.014” stainless steel (SS) archwires (Highland Metals Inc., Franklin, IN, USA), delivering light continuous forces; visits were scheduled every 4 weeks under a single lead operator’s supervision. Periapical radiographs were obtained every 6 months to monitor root position. Once adequate interradicular space between U2 and U1 was visible, second-order bends were added to create a 1 mm space between U2 distal root and IPU3 cusp. For narrow interradicular spaces, angulated brackets were applied to U1 to facilitate movement. Conversely, in cases where U1 movement was not intended, potential reciprocal side effects related to reaction forces, including unintended U1 movement or occlusal changes, were monitored throughout treatment using periodic clinical and radiographic evaluation.

### 2.3. OPG Analysis

**Figure 1:**
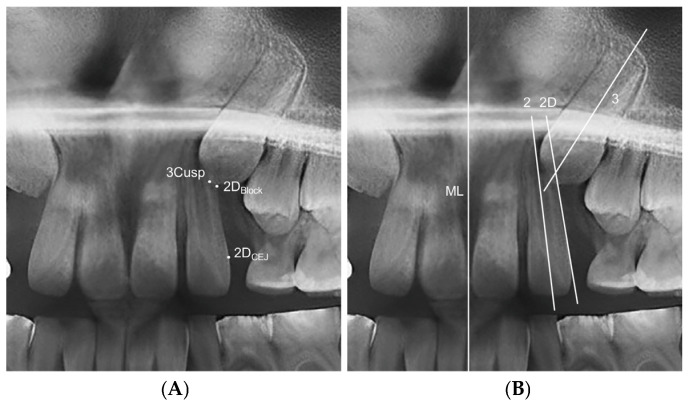
Panoramic radiographs showing: (**A**) reference points; (**B**) reference lines. Detailed descriptions of all reference points and lines are provided in Table 1.

**Figure 2:**
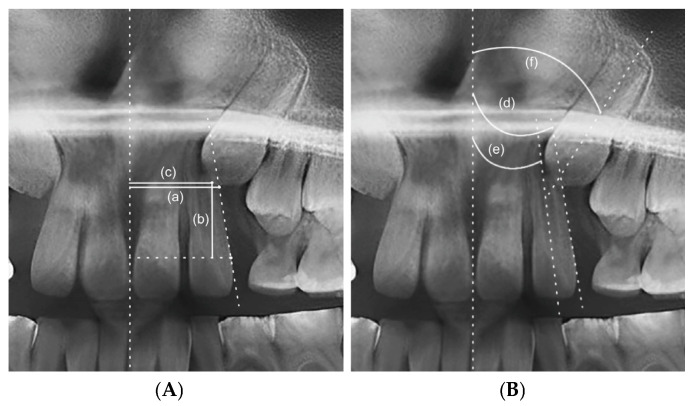
Panoramic radiographs showing: (**A**) Linear measurements (solid lines): (a) 2DBlock-ML (2DHorz), (b) 3Cusp-2DCEJ (3Vert), and (c) 3Cusp-ML (3Horz); (**B**) Angular measurements (solid lines): (d) 2D^ML, (e) 2^ML, and (f) 3^ML. Detailed descriptions of all measurements are provided in Table 2.

A single independent investigator, calibrated by a Thai board-certified orthodontist with over 20 years of experience, performed all OPG measurements. Reference points, lines, and linear/angular measurements are detailed in Table 1 and Table 2 and Figure 1 and Figure 2.

### 2.4. Statistical Analysis

Reliability was assessed using intraclass correlation coefficients (ICCs) and measurement error via Dahlberg’s formula to evaluate measurement reproducibility and potential measurement errors. Normality was evaluated using the Shapiro–Wilk test (*p* > 0.05). Paired t-tests compared positional changes (T1–T0), Pearson’s coefficients evaluated variable relationships, and multiple linear regression identified predictors of 3Vert among U2 parameters. Multicollinearity among independent variables was assessed using variance inflation factor (VIF) analysis. Regression assumptions were evaluated through residual diagnostics. Analyses were performed using SPSS software v26 (IBM Corp., Armonk, NY, USA), and statistical significance was set at *p* < 0.05.

## 3. Results

This study included 29 patients (18 females, 11 males) aged 10–12 years, with a mean age at T0 of 10.6 ± 0.6 years. The mean follow-up duration (T1–T0) was 12 ± 1 months (range, 11–13 months). A total of 37 IPU3 (eight bilateral and 21 unilateral cases) were analyzed. All U3s were initially located in sector II based on the method of Ericson and Kurol [18]. Measurement reliability was good to excellent, with a mean ICC of 0.86 (range, 0.82–0.90). Measurement errors, assessed using Dahlberg’s formula, were 0.32 mm for linear and 0.85° for angular variables, both within acceptable orthodontic thresholds (<0.5 mm and <1°, respectively) [20].

Mesial root tipping of U2 produced significant positional changes in IPU3. Representative panoramic radiographs at T0 and T1, demonstrating eruption of the IPU3 following mesial root tipping of the U2. The mean mesial U2 root movement away from the U3 was 2 mm (2DHorz), with mean U2 distal root surface angulation of 5.6° (2D^ML) and long axis tipping of 6.3° (2^ML) (Table 3). Horizontal mesial root movement (2DHorz) correlated with mesial root tipping (2D^ML, 2^ML) (Table 4). IPU3 exhibited a mean vertical movement (3Vert) of 3.7 mm, mean reduction in angulation (3^ML) of 7.5°, and 2.1 mm distal cusp tip shift (3Horz) (Table 3).

Pearson correlation analysis revealed a strong positive association between 3Vert and 2DHorz (r = 0.697, *p* < 0.001), and moderate negative correlation with 2D^ML (r = −0.451, *p* = 0.006) and 2^ML (r = −0.416, *p* = 0.013). Most U2 mesial root parameters were associated with U3 positional changes, except for 2^ML/3^ML and 2^ML/3Horz (Table 4).

Multiple linear regression analysis with 3Vert as the dependent variable and 2DHorz, 2D^ML, and 2^ML as predictors, indicated a significant model (R = 0.714, R^2^ = 0.51, *p* < 0.001). Multicollinearity analysis demonstrated acceptable VIF values (<5) for all predictors, indicating no significant multicollinearity among independent variables. Among predictors, only 2DHorz was a significant positive predictor of 3Vert, revealing that greater mesial movement of 2DBlock was associated with increased 3Vert. Neither 2D^ML nor 2^ML showed significant effects (*p* > 0.05) (Table 5).

## 4. Discussion

This study focused on the vertical eruption of IPU3 (3Vert). Unlike previous studies [18,21] that measured vertical eruption relative to the occlusal plane, this study measured the distance from the 3Cusp tip to the cemento-enamel junction (CEJ) of the adjacent U2 root parallel to the midline. The midline served as a stable reference unaffected by head tilt, whereas the occlusal plane may vary with head position [22]. Measuring to the CEJ provides a reliable indicator of eruption, as a reduction in 3Cusp-to-CEJ distance reflects vertical tooth movement despite potential changes in the occlusal plane owing to leveling or wear [23].

Horizontal U2 movement (2DHorz) was assessed using 2DBlock, the contact point obstructing IPU3. Releasing this contact facilitates U3 eruption along the distal root of U2 [24]. Although previous studies [25,26,27] evaluated U2 width, length, and angulation to predict IPU3, none directly measured horizontal displacement relative to the midline. This parameter may serve as a meaningful indicator for treatment planning.

A novel parameter (2D^ML), representing the angulation of the U2 distal surface and in contact with IPU3, was introduced. Unlike traditional 2^ML, which measures U2 long axis [28,29], 2D^ML captures the actual contact interface with the canine. To the best of our knowledge, no prior studies are available for direct comparison. Therefore, this parameter should be considered exploratory and requires further external validation in future studies.

At T0, mean 2^ML averaged 8.3 ± 3.3°. Approximately 75% of U2s exhibited lower angulation than those in normally erupting canines, which is consistent with prior findings that upright U2 increases the risk of IPU3 [17,30]. During the “ugly duckling” stage, U3 typically erupts along the distal surface of U2, producing distal crown tipping [15]. Upright U2 deviates from this natural trajectory, thereby highlighting its potential role in impaction at approximately 10 years of age [16].

All U3s were located in sector II, the most frequently observed sector in Lindauer et al. [31] Sector II positioning predicts a 64% likelihood of spontaneous eruption following deciduous canine extraction [18]. However, with fully developed and erupted U2 overlapping the U3, spontaneous eruption probability of normal U3 drops significantly to 7–11% [19]. In this study, these conditions justified the initiation of treatment. Although direct comparison was limited by the absence of an untreated control group, the favorable eruption outcomes observed in all treated cases contrast with the relatively low spontaneous eruption rates previously reported in similar clinical situations [19]. In addition, the mean U3 angulation at T0 (3^ML) was 21.9 ± 3.5°, exceeding values reported for normally erupting U3s [17,29]. Notably, this 3^ML value surpassed the impaction predictive threshold of 14.1° [32]. Furthermore, this aligns with meta-analysis findings indicating that angulations above 19.9° strongly indicate IPU3 [25].

Following this radiographic assessment, all patients were observed for 6 months to monitor spontaneous U3 eruption. No positional changes were detected, confirming that initiating active treatment at T0 was appropriate. Based on predictive parameters and the absence of palpable U3, all included U3s were considered at high risk of impaction.

U3 angulation (3^ML) at T1 averaged 14.4 ± 3.4°, approaching the normal eruptive angulation range [25], indicating that U2 adjustment can guide the U3 toward a more upright position, consistent with the normal occlusal development [16].

This study evaluated the relationship between U2 positional changes, measured via 2DHorz, 2D^ML, and 2^ML, and U3 outcomes including 3Vert, 3^ML, and 3Horz. The primary outcome, 3Vert, represents successful eruption, and was measured as the movement of the cusp tip toward the alveolar ridge and approximated by the CEJ of the adjacent U2.

Mesial movement of 2DBlock was achieved through mesial root tipping of the U2, represented by both 2D^ML and 2^ML. Although these two parameters strongly correlated with each other (r = 0.854), 2D^ML exhibited a stronger correlation with U3 positional changes than did 2^ML, whereas 2^ML did not show a statistically significant relationship with either 3^ML or 3Horz. These findings reveal that canine eruption is more strongly influenced by the proximity of the distal root surface of the U2 (as represented by 2D^ML) than by changes in the overall long-axis angulation of the tooth (2^ML).

Nevertheless, previous studies evaluating U2 morphology and inclination have reported inconsistent findings regarding their predictive value for canine impaction [2,25,30]. These discrepancies may reflect differences in imaging methods, patient age ranges, eruption stages, and the specific radiographic parameters evaluated. Unlike studies focusing primarily on overall U2 angulation, the present study specifically evaluated the distal root contact area interacting with IPU3.

The strongest correlation with 3Vert was observed at 2DHorz, indicating that horizontal mesial repositioning of the U2 root (2DBlock) and reduction of overlap with U3 significantly facilitated natural eruption of IPU3. Regression analysis confirmed 2DHorz as the only significant predictor of 3Vert, thereby highlighting the predominant role of horizontal root movement in promoting U3 eruption.

Although the observed positional changes were relatively small in magnitude, even limited mesial U2 root movement may still be clinically meaningful during early interceptive treatment because canine eruption is sensitive to interradicular spatial relationships during the mixed dentition stage. Previous studies have shown that relatively small positional differences on panoramic radiographs may influence eruption outcomes and impaction risk assessment [33]. Therefore, minor changes in U2 root position may still contribute to clinically meaningful modification of the eruption pathway.

Although the regression model explained approximately 51% of the variance in 3Vert, it only reflected U2 positional changes. The remaining unexplained variability suggests that canine eruption is influenced by multiple biological, developmental, skeletal, genetic, and three-dimensional spatial factors beyond U2 positional changes alone [34]. Variability in craniofacial growth patterns, eruption timing, periodontal environment, and individual biological response may also contribute to differences in eruption outcomes.

Overall, the findings emphasize that repositioning U2 away from U3 plays a more critical role in facilitating eruption than modifying U2 angulation alone. Clinically, mesial root tipping is preferred over bodily movement because of greater available alveolar bone apically, allowing safer and more effective repositioning.

Although all included cases demonstrated favorable eruption outcomes, the present findings may not be applicable to more severe impaction patterns or biologically unfavorable conditions. Potential clinical situations in which mesial U2 root tipping alone may be insufficient include severe canine displacement (e.g., sectors III–V), unfavorable three-dimensional canine position, ankylosis, inadequate arch space, or advanced skeletal discrepancies. These factors should be carefully evaluated during treatment planning, and future studies including unsuccessful cases are needed to better define prognostic indicators and clinical limitations of this approach.

### 4.1. Recommended Treatment and Monitoring

The findings of this study recommend mesial angulation of the U2 root by approximately 5.6° at the distal surface or 6.3° along the long axis, resulting in a mesial horizontal movement of 2 mm. This approach may be effective in initiating eruption of IPU3 cases with a non-palpable canine bulge, U3/U2 overlap, and U3 positioned in sector II on OPG, as classified by Ericson and Kurol [18]. Consistent radiographic monitoring is advised to evaluate U3 eruption and verify ongoing treatment effectiveness.

### 4.2. Limitations

This study included a sample size of 37 IPU3 based on prior sample size calculations; a larger sample would improve control over confounding, enhance generalizability, and improve the stability of regression-based analyses. In addition, potential confounding variables such as sex, facial pattern, and dental development stage were not separately analyzed or statistically controlled, which may have influenced the observed associations. The absence of a control group, owing to ethical considerations, limits comparison with untreated cases, which may remain impacted or erupt spontaneously, potentially increasing the risk of adjacent U2 root damage, eruption through non-keratinized gingiva, or palatal impaction [12].

Because only sector II IPU3 cases were included, caution is needed when generalizing these findings to different clinical presentations.

Furthermore, this study included only successfully erupted IPU3 cases, which may have introduced selection bias and limited the internal validity of the findings. Therefore, the observed associations between U2 root positional changes and IPU3 eruption should be interpreted with caution and may not be generalizable to unsuccessful or untreated cases. The relatively low variability observed in several measurements may also reflect the intentionally homogeneous sample resulting from the strict inclusion criteria applied in this study. Because bilateral cases were included, complete statistical independence between observations could not be assumed, as teeth from the same patient may share developmental and biological characteristics, which should be considered when interpreting the findings.

The exclusive use of OPG may limit three-dimensional accuracy compared with CBCT imaging in impacted canine assessment because of inherent distortion and magnification errors that may affect linear and angular measurements [35]. Nevertheless, serial panoramic radiographs allowed standardized longitudinal evaluation with minimized radiation exposure, as panoramic measurements remain clinically useful for longitudinal assessment of impacted canines [33]. Therefore, CBCT evaluation may be considered in cases with suspected severe displacement, unclear buccopalatal localization, or complex root relationships, where precise three-dimensional assessment is necessary before initiating root tipping mechanics [36].

The mean follow-up period of 12 months captured early eruptive changes. Further longitudinal follow-up until the IPU3 reaches the final occlusal plane would provide additional insights into long-term stability, complete eruption outcomes, and self-adjustment mechanisms.

## 5. Conclusions

Mesial root tipping of the U2 was associated with horizontal mesial root movement and favorable eruption changes in IPU3 in early adolescent patients. These findings were observed specifically in cases with a non-palpable IPU3 in sector II and a fully developed U2 root. However, these findings should be interpreted as associations rather than evidence of causality.

## Figures and Tables

**Table 1:** Reference points and lines.

Symbol	Description
2D_CEJ_	Distal cemento-enamel junction (CEJ) of the U2
2D_Block_	Blockage point on the distal root surface of U2 at the most incisal contact where it overlaps U3. For T1, where the overlap is absent, a reference line from 2D_CEJ_ to 2D_Block_ in T0 image is transposed onto T1; the end of this line represents 2D_Block_ location. This approach was used to maintain consistency of landmark identification between T0 and T1 images and to improve reproducibility of longitudinal measurements.
3Cusp	U3 cusp tip
ML	Midline through the anterior nasal spine and central incisors
2D	Distal root surface line of the U2
2	Long axis of the U2
3	Long axis of the U3

Abbreviations: U2, maxillary lateral incisor; U3, maxillary canine; T0, timepoint at treatment initiation; T1, 1-year follow-up after treatment initiation.

**Table 2:** Parameter measurements.

Symbol	Description
U2 position 1. Horizontal	
• 2D_Block_-ML (2DHorz)	Perpendicular distance from 2D_Block_ to ML
2. Angulation	
• 2D^ML	Angle between 2D and ML
• 2^ML	Angle between 2 and ML
U3 position	
1. Vertical	
• 3Cusp-2D_CEJ_ (3Vert)	Vertical distance between two horizontal lines perpendicular to ML and tangent to 3Cusp and 2D_CEJ_
2. Angulation	
• 3^ML	Angle between 3 and ML
3. Horizontal	
• 3Cusp-ML (3Horz)	Perpendicular distance from 3Cusp to ML

**Table 3:** Lateral incisor and canine positional changes at T0 and T1.

Variable		Mean	SD	*p* Value	Mean Difference	95% CI	
						Lower Limit	Upper Limit
2DHorz	T0	16.6	0.9	<0.001 **	−2.0	−2.2	−1.7
	T1	14.6	0.6				
2D^ML	T0	7.0	1.4	<0.001 **	5.6	5.0	6.1
	T1	12.6	1.5				
2^ML	T0	8.3	3.3	<0.001 **	6.3	5.5	7.0
	T1	14.6	2.9				
3Vert	T0	9.9	1.7	<0.001 **	−3.7	−3.9	−3.4
	T1	6.2	1.4				
3^ML	T0	21.9	3.5	<0.001 **	−7.5	−8.0	−7.0
	T1	14.4	3.4				
3Horz	T0	17.6	1.7	<0.001 **	2.1	1.8	2.4
	T1	19.7	1.7				

Note: The paired t-test was used to compare positional changes between T0 and T1. Note: 2DHorz, 3Vert, and 3Horz were measured in millimeters (mm). Conversely, 2D^ML, 2^ML, and 3^ML were measured in degrees (°). ** Significant at *p* < 0.01. Abbreviations: CI, confidence interval; SD, standard deviation.

**Table 4:** Pearson correlation coefficients (r) between lateral incisor and canine positional changes.

Variable	2DHorz	2D^ML	2^ML	3Vert	3^ML	3Horz
2DHorz	1.000					
2D^ML	−0.405 *	1.000				
2^ML	−0.451 **	0.854 **	1.000			
3Vert	0.697 **	−0.451 **	−0.416 *	1.000		
3^ML	0.656 **	−0.428 *	−0.307	0.668 **	1.000	
3Horz	−0.561 **	0.394 *	0.331	−0.606 **	−0.578 **	1.000

Note: Pearson’s correlation coefficient was used. * Significant at *p* < 0.05. ** Significant at *p* < 0.01.

**Table 5:** Multiple linear regression analysis predicting canine vertical eruption (3Vert) using lateral incisor positional changes.

Independent Variable	Unstandardized Coefficient	*p* Value
Constant	−1.871 **	<0.001
2DHorz	−0.666 **	<0.001
Adjusted R^2^ = 0.51		
Regression equation: 3Vert = −1.871 − (0.666 × 2DHorz)		

Note: Multiple linear regression analysis was performed with 3Vert as the dependent variable. ** Significant at *p* < 0.01. Abbreviation: Adjusted R^2^, adjusted coefficient of determination.

## Data Availability

The data presented in this study are available upon request from the corresponding author.

## Abbreviations

The following abbreviations are used in this manuscript:

CBCT	Cone-beam computed tomography
CEJ	Cemento-enamel junction
ICC	Intraclass correlation coefficient
IPU3	Impacted maxillary canine
ML	Midline through the anterior nasal spine and central incisors
NiTi	Nickel-titanium
OPG	Orthopantomogram
R^2^	Coefficient of determination
SS	Stainless steel
T0	Treatment initiation
T1	One-year follow-up after treatment initiation
U2	Maxillary lateral incisor
U3	Maxillary canine
2	Long axis of the U2
2D	Distal root surface line of the U2
2D_CEJ_	Distal cemento-enamel junction of the U2
2D_Block_	Blockage point on the distal U2 root
2DHorz	Perpendicular distance from 2D_Block_ to ML
2D^ML	Angle between 2D and ML
2^ML	Angle between 2 and ML
3	Long axis of the U3
3Cusp	U3 cusp tip
3Vert	Vertical distance between horizontal lines tangent to 3Cusp and 2D_CEJ_
3Horz	Perpendicular distance from 3Cusp to ML
3^ML	Angle between 3 and ML
